# Smart Contract-Based Review System for an IoT Data Marketplace

**DOI:** 10.3390/s18103577

**Published:** 2018-10-22

**Authors:** Ji-Sun Park, Taek-Young Youn, Hye-Bin Kim, Kyung-Hyune Rhee, Sang-Uk Shin

**Affiliations:** 1 Interdisciplinary Program of Information Security, Graduate School, Pukyong National University, Busan 48513, Korea; 201211812@pukyong.ac.kr (J.-S.P.); khbin1346@pukyong.ac.kr (H.-B.K.); 2 Electronics and Telecommunications Research Institute, Daejeon 34129, Korea; taekyoung@etri.re.kr; 3 Department of IT Convergence and Application Engineering, Pukyong National University, Busan 48513, Korea; khrhee@pknu.ac.kr

**Keywords:** IoT, data marketplace, blockchain, Ethereum smart contract, smart home, home automation, data integrity

## Abstract

Internet of Things (IoT)-based devices, especially those used for home automation, consist of their own sensors and generate many logs during a process. Enterprises producing IoT devices convert these log data into more useful data through secondary processing; thus, they require data from the device users. Recently, a platform for data sharing has been developed because the demand for IoT data increases. Several IoT data marketplaces are based on peer-to-peer (P2P) networks, and in this type of marketplace, it is difficult for an enterprise to trust a data owner or the data they want to trade. Therefore, in this study, we propose a review system that can confirm the reputation of a data owner or the data traded in the P2P data marketplace. The traditional server-client review systems have many drawbacks, such as security vulnerability or server administrator’s malicious behavior. However, the review system developed in this study is based on Ethereum smart contracts; thus, this system is running on the P2P network and is more flexible for the network problem. Moreover, the integrity and immutability of the registered reviews are assured because of the blockchain public ledger. In addition, a certain amount of gas is essential for all functions to be processed by Ethereum transactions. Accordingly, we tested and analyzed the performance of our proposed model in terms of gas required.

## 1. Introduction

**Figure 1:**
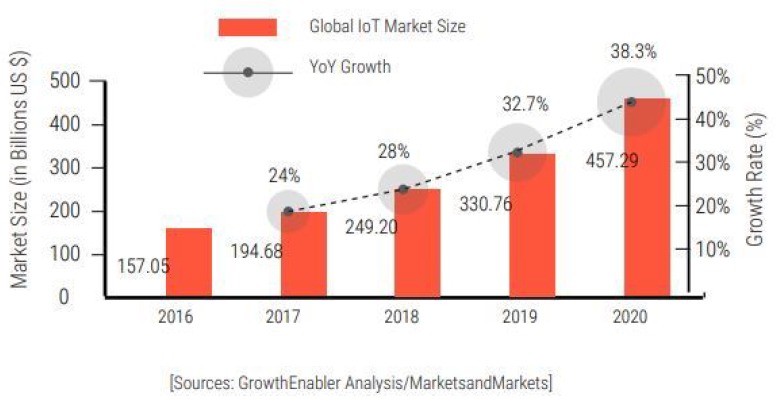
IoT market size and growth forecast [6].

Nowadays, the term “ubiquitous computing” is widely known, which refers to a computing environment that a user can access through any device with no restrictions in terms of time and place [1]. For a long time, the research on ubiquitous computing has progressed steadily. In 1966, the first ubiquitous computing study investigated wearable computing and attempted to combine clothing with computers [2]. Ubiquitous computing is a broader term; thus, it can be classified into various sub-topics. One of them is called the Internet of Things (IoT), which has gained considerable attention in recent years. The IoT refers to a network environment, in which a large number of objects, sensors or devices are connected through the Internet or some other communication infrastructure to provide value-added services [3]. This term was first coined in 1998, and the basic idea behind this technology is to connect people and objects anywhere and anytime [4]. In the near future, we will live in a society where all objects surrounding us will be smart objects interconnected via the IoT, communicating with each other with minimal human intervention. Thus, the ultimate objective of the IoT is a better world, where every device is aware of our desires and needs and acts on behalf of human beings without requiring explicit instructions [5]. Consequently, the market for IoT devices has been continuously expanding. As shown in Figure 1, GrowthEnabler forecasts that the global IoT market scale will reach $457.2 billion by 2020 [6].

Smart homes are a representative application field of the IoT, which are being investigated by researchers to enhance people’s living comfort. Smart homes or home automation, in which appliances interact with each other as smart objects, are/is one of the many IoT platform. In such applications, a home’s safety, temperature, humidity and energy are controlled by a home automation system, which the residents can inspect in real time by smart phone applications. Moreover, emerging home appliances combined with the artificial intelligence technology can learn and analyze the behavioral patterns of users to improve home automation further. Various smart home devices focus not only on electronic equipment features, but also on people’s daily activities and healthcare monitoring [7]. For example, such devices can perform health monitoring tasks, such as checking blood pressure in real time and transmitting data to the doctor if a health issue is detected. More enterprises are attempting to enter IoT smart home field because of the high market potential of the IoT-based smart homes.

A common characteristic of most smart objects in a smart home is that they accumulate log data obtained through sensors during operation. If a change is detected in a sensor’s value at a predetermined interval, the devices share this value with the user. Most smart home device companies are interested in this large amount of data. This is because it is possible to observe user behavior patterns or detect errors in a system through a large amount of log data analysis stored in such devices. Based on these data, a better IoT solution can be developed or updated and presented to the user. As competition in the IoT home automation market becomes more intense, companies need to provide consumers with more convenient and efficient IoT solution applications, as well as device performance. Most smart devices are used in conjunction with smartphones. The various OS versions in smartphones and the IoT solution applications must be well tested, and the log data gathered from various users are essential. It can also be used to develop new products that target a specific age group using log data collected from users of a certain age. However, companies are required to obtain the user’s consent before sharing their data because of privacy concerns and other issues. In the past, companies traded user data on their private trading platform. However, as the size of the IoT market grows, the size of the data trading platform is also increasing. Recently, this platform was named the data marketplace and, peer-to-peer (P2P) distributed networks have been widely used to facilitate trading on this platform, which ensure that users and enterprises are equal participants in the network.

Although a P2P-based data marketplace has the advantage of all participants being equal [8], the participants engage in financial transactions without establishing a trusted relationship. As data consumers, companies cannot completely trust sellers in a data marketplace. Thus, if reviews of the available data or sellers are submitted by the previous buyers, a potential buyer can consult them to make a more rational decision. However, on the existing server-client-based review systems, the server administrator is able to modify or delete the reviews. Thus, the existing review systems cannot ensure the integrity of all reviews to every user of the system.

In this study, we propose and analyze a novel review system. The proposed system confirms the quality of data traded in the P2P data marketplace and the reputation of the data seller before a transaction occurs. To achieve this, we use an Ethereum smart contract as the core element of the proposed system. Ethereum is a blockchain platform; thus, it ensures not only the integrity and immutability of the data, but also the transparency of the entire system. The main users of the smart contract review system are IoT device manufacturers, who can register and check reviews for the data that they wish to purchase by visiting a web page. In Section 2, we discuss studies on data marketplace and Ethereum smart contracts in detail. In Section 3, we discuss the proposed system’s structure, the process of registering a review and the Solidity code used in our review system. In Section 4, we compare features of the server-client review model and the model proposed in this study. Subsequently, we analyze and evaluate the performance of the proposed system. Finally, we present the conclusions drawn from this study in Section 5.

## 2. Related Work

### 2.1. IoT Data Marketplace

In modern society, information is a strategic asset that can secure and maintain competitiveness. An enterprise can benefit by developing better products or by providing services based on a large amount of information. Especially, usage log data are essential for companies whose major products are IoT devices, because these companies use log data generated by devices to obtain a statistical representation of user behavior with the objective of discovering a product’s shortcomings, or to complement the existing products by developing new ones [9]. Many studies have described the IoT data sharing model as a sensing as a service ($S^{2}\mathrm{aaS}$) model [10].

In the past, enterprises could transfer raw IoT data acquired by users or obtained from data brokers, to a cloud server. However, this has caused serious concerns with regard to user privacy. Consequently, the data marketplace emerged as a platform to facilitate transparent data transactions between IoT device users and IoT device producers. The participants in a typical data marketplace are largely divided into data consumers and data sellers. Data sellers sell their data for economic purposes or to build trust, and data consumers purchase and use these data. If necessary, there may exist a data intermediary capable of analyzing IoT log data to produce secondary processed data. Thus, according to this model, those who own and sell data become users of the IoT devices, while data consumers become data producers. Therefore, a data owner is incentivized to sell the user-generated device data in the marketplace, and a data consumer can acquire high-quality data that fulfill their requirements [11].

The P2P-network-based data marketplace is a more fair system as compared to a system where all transactions are handled by a central server. Moreover, there is no single point of failure (SPOF); therefore, in the proposed system, there is less risk of the entire system failing [12]. In a P2P network, all peers on the network communicate with equal privileges, i.e., the system has the advantage of being a distributed network. The data owner uploads the data and promotes them to other data consumers on the marketplace by using the P2P network. Most $S^{2}\mathrm{aaS}$ models use cloud servers because the transactions require a large database server for various types of data sharing. The data owner uploads all the encrypted raw data on the cloud server. Further, the data owner reveals only the metadata or hash value to the consumer and provides a key (e.g., decryption key) that provides access to the cloud server’s raw data once the transaction is confirmed. However, the shortcoming of this P2P marketplace model is that buyers cannot fully trust the shared data because they decide whether they want to trade based on the available metadata only.

Every online commerce platform has the problem of trust in the merchandise and the seller that it deals with, and the same goes for the data marketplace [13]. This is because consumers and sellers do not directly meet and do business. To redeem the absence of seller trust information, a reputation system or a review system is required. A reputation or a review system allows buyers who have attempted a transaction before to leave an evaluation of the goods and sellers. Furthermore, although the buyer has not used the goods beforehand, he/she can predict whether the seller can be trusted based on the reviews. The review system is even more essential in a P2P network-based platform. In a server-centralized network, a server can manage the network by using identity information of the seller and consumer. In a P2P network, however, all peers act anonymously without disclosing their identity information. By exploiting this point, network attacks can be performed on other peers or fraudulent transactions can be made with fake identity information [14]. In the P2P network of the IoT data marketplace, the economic loss caused by transaction frauds can be very large because various companies participate. As a result, IoT companies that are involved in a transaction can avoid the worst situation based on the reputation that is honestly written about the seller and the IoT data that are traded. After, one can leave a review for other consumers about the transaction results.

### 2.2. Ethereum Smart Contracts

The blockchain technology, on which Bitcoin is based, has become a popular topic since the advent of Bitcoin in 2008 [15]. In brief, a blockchain is a decentralized database. A data unit, which is called a transaction, is collected by a miner node and combined with other transactions in a block [16]. A cryptographic hash function is used to link the current block with a previous one like a chain. All transactions and blocks on the chain are transparent to the network nodes; thus, the blockchain is also referred to as a public ledger. Moreover, the blockchain adopts a P2P network running without a third trusted party or a centralized server, which can be an SPOF. Several nodes on the network possess a copy of this public ledger. If a malicious node wants to change the data stored in the ledger, it must change the data stored in the public ledger copies held by each node. Additionally, when the data of one block changes, the chain structure causes the hash value of that block to change, as well, and consequently, the hash values of the child blocks connected to the changed block also change. Therefore, any change in the blocks can be immediately detected by all nodes. Thus, the blockchain system ensures the integrity, consistency and transparency of the data stored in the chain. On a P2P network, where a trust relationship between peers does not exist, a consensus algorithm is required to deal with various important issues, such as block generation authority. The blockchain research to expand the range of blockchain applications beyond cryptocurrency is ongoing and is regarded as the primary technology of the fourth industrial revolution globally.

Ethereum is a distributed virtual machine that can execute smart contracts defined by a developer as a Bitcoin extension protocol [17]. When Ethereum was first developed in 2013, Vitalik Buterin proposed the extension of the Turing-incomplete Bitcoin script to a Turing-complete language capable of handling various types of smart contracts. Turing-completeness [18] facilitates the development of cryptocurrency, smart contracts and other decentralized applications (Dapps) by implementing various blockchain platforms [19]. In Bitcoin, the unspent transaction output (UTXO) is simply expressed as spent/unspent; whereas, Ethereum represents everything as the state transformation of the blockchain or an account. Ethereum’s smart contracts consider an account as a normal node. Therefore, a node is able to send coins to a smart contract account and receive coins from that account. In Ethereum, the currency used is called Ether or Wei. Moreover, Ethereum uses a special programming language called Solidity. This language is a Turing-complete bytecode language called the Ethereum virtual machine bytecode and can use arrays, variables, constructors, etc., in the same manner as any typical Turing-complete programming language. The functions define the specific operation of these elements, and several types of Dapps, such as web sites or smartphone apps, can be developed using Ethereum smart contracts. Compared to Bitcoin, Ethereum has the advantageous features of scalability and flexibility.

(1) transactionfee=gasprice×gaslimit

Gas is a characteristic element of Ethereum platform. Specifically, gas is to be considered while dealing with the cost of storage space or the computational power required to run smart contract functions, run the code, and so on [19]. All operations are done on the network, such as deploying smart contracts or sending coins, creating transactions. The transaction fee is calculated by (1), and the amount of gas required to execute a code is defined in the Ethereum yellow paper [17]. If the gas price is high, the transaction has a high reward and will probably be mined relatively early. The creator of the transaction can set a gas limit to prevent too much gas from being consumed during the execution of a smart contract code. In Ethereum, the transaction creator node always pays the gas cost.

## 3. Proposed Review System

On a data marketplace platform, an enterprise, which is primarily the data consumer, analyzes the data shared by the data owner and uses the analysis results. Such operations lead to the development of new IoT solutions or new products. Data transactions are very important to data consumers. However, it is necessary to verify the data owner and data quality before sharing any data on the marketplace because of the characteristics of the P2P decentralized model, which does not form perfect trust between the peers. Additionally, only the metadata or data hash values are disclosed because of the storage space requirements and security concerns, and the actual data are not available directly in the data marketplace. Once the transaction is confirmed, the data owner provides the consumer with a key to access the data stored in an external database, such as a cloud database. If a review system is available to review the data being traded, it is possible to check the reputation of the data owner and decide whether the data being sold are reliable based on the submitted reviews. Written reviews are influential; thus, the integrity of a written review should be ensured. Additionally, someone might search through reviews that were submitted long ago. Therefore, the immutability of the reviews needs to be guaranteed. Consequently, in this study, we developed a blockchain-based review system to ensure the immutability, integrity and reliability of the data. In particular, among the various blockchain platforms, the Ethereum smart contracts can be used effectively in this system because they do not only provide a simple cryptocurrency function, but also provide smart contracts that enable other types of functionality. Thus, we created a smart contract with the Solidity language and used it to develop a Dapp that manages reviews. Additionally, we combined it with a web page written in HTML.

In Section 3.1, we discuss the structure of the smart contract-based review system for IoT data available on the IoT data marketplace, as well as the system’s operating procedure. The Solidity code is presented in Section 3.2.

### 3.1. Overall System Architecture

**Figure 2:**
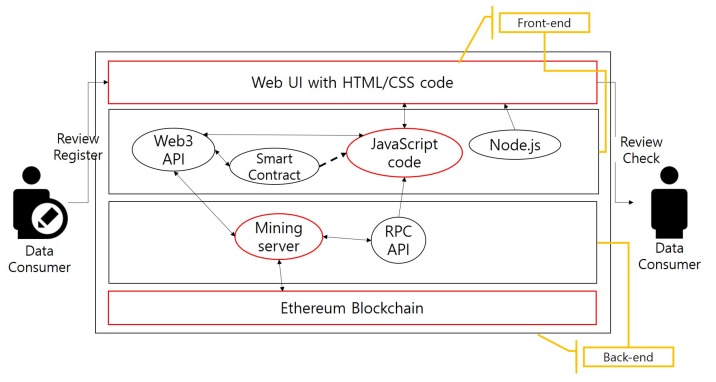
System architecture.

**Figure 3:**
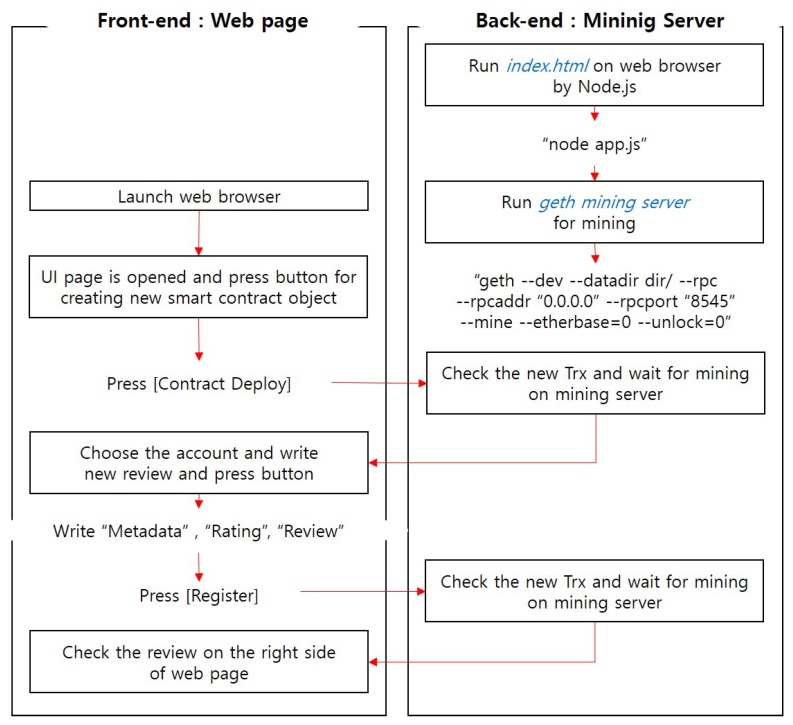
Function process of “setReview()”.

The review system for the IoT data has four components: a web browser, which is the user interface (UI), the JavaScript code containing the smart contract bytecode, a mining server receiving the generated transactions within a block and an entire blockchain database (as shown in Figure 2). Figure 3 shows the review registration process, in which these components operate.

First, the JavaScript code component shown at the center of Figure 2 performs several functions. This code is a front-end component linked to the HTML code of the web page. The smart contract’s application binary interface (ABI) and the bytecode values are referenced by the JavaScript code. To use the data structures and functions defined in the smart contracts, a new smart contract is created using the ABI and bytecode values. Further, this smart contract communicates with the mining server through the Web3 application programming interface (API). Many functions that create transactions by calling the appropriate functions of the smart contract are defined in the JavaScript code. These functions receive the values from the HTML page, use them as the transaction’s input values and deliver the output result when the block is mined successfully.

A web page is a front-end element interacting directly with the user. In this study, a web page was developed using the HTML and CSS code and was connected to a web browser, which is an HTTP interface, using Node.js. When the user adds a review, checks a registered review or modifies a written review, a function of the corresponding JavaScript code is called. As shown in Figure 3, the review writer launches the web browser, and then, the linked UI page is opened. On the web page, the “Contract Deploy” button is clicked to create a new smart contract, and the user selects one of their accounts to be used as the writer’s identity of the review. A unique value is entered for the shared data (metadata); a rating is given; and a brief review is submitted. To check the review, a user can select the account that he/she created and enter the metadata value to confirm the submitted rating and review. A review can be modified in a manner similar to registration, in which the user enters the metadata, modified rating and modified content. Recently added or edited reviews are shown at the right side of the main web page. The detailed implementation result is described in Section 4.2.

For the block creation step, which is a key task on the blockchain platform, a mining server is operated. The transaction generated after calling the function requested by the user on the web page must be included in the block through mining to reflect the result. Detailed mining operation commands are shown in Figure 3. The mining server is run by the geth interface. The Ethereum geth interface can use JSON remote procedure call (RPC) protocol; therefore, it uses the HTTP-based RPC API on port 8545. Moreover, “etherbase,” refers to the account for which a block reward will be paid by mining, and “- -etherbase = 0” means that account[0] is an etherbase account. The “- -unlock = 0” option can be added to unlock the default lock on the Ethereum account and submit the transaction. As soon as the “sendTransaction()” code, which calls the smart contract function through the JavaScript code, is executed, the transaction hash value and the process can be checked in the mining server window. A certain amount of time is required until a transaction is mined to a block.

### 3.2. Smart Contract Code

Ethereum smart contracts use a particular contract-oriented programming language called Solidity, which was first developed by Gavin Wood [20]. The platform can be developed using a tool called Remix Solidity IDE, which enables the deployment and execution of smart contracts virtually. Additionally, to use the smart contract-based Dapp, we implemented the Web3 API and a value defining the architecture of the smart contract. In the proposed model, the ABI and bytecode values are referenced by the JavaScript code, and the smart contract functionality and structure are used.

**Figure 4:**
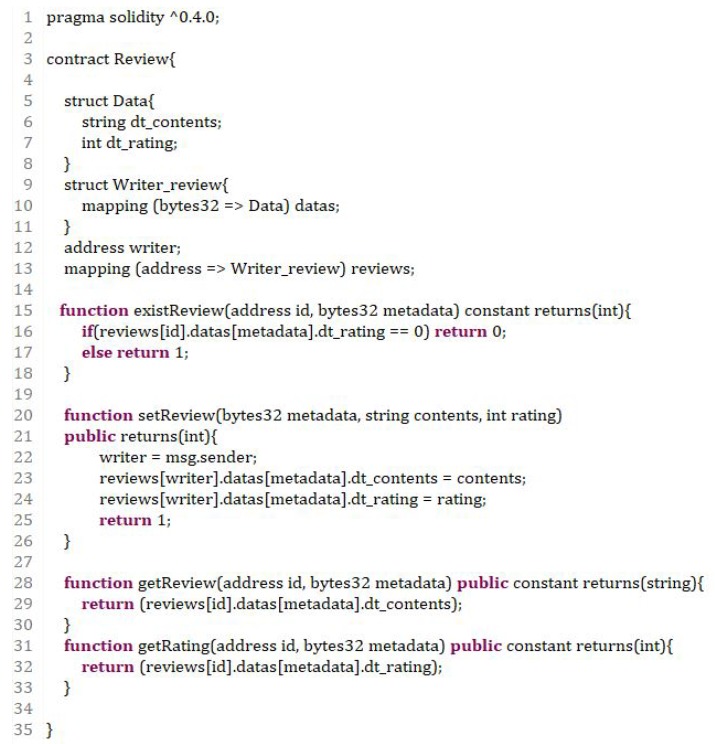
Solidity code for the review system.

The smart contract used in this review model consists of four functions and two structures, as shown in Figure 4. Each of the four functions performs operations to check for the existence of a review, register a review, check the content of a review or check the ratings associated with a review. In the two structures, the “Data” structure for storing the reviews consists of “dt_contents,” which is a string value, and “dt_rating,” which is the rating associated with a review. The “Writer_review” structure contains “datas,” an array of “Data” structures, indexed by the metadata values with the “mapping” keyword. Additionally, a user can look at the reviews, stored in the “Writer_review” structure array, created by each address using the address value of the transaction creator as an index.

The “setReview()” function, which functions as a review subscription, takes the metadata values, data quality details and ratings as the input values. This function is used by consumers who have previously purchased data from the data marketplace to review the quality or mention the pros and cons of the data. The metadata input item is a value computed by the SHA256 cryptographic hash function in the JavaScript code. The cryptographic hash function yields a significantly different output value, even if the input is different by only one bit. Thus, each item can be easily distinguished in the JavaScript code and smart contract structures. The rating input value is rated higher for data that are recommended to other consumers. The content input value is a variable length variable value, which can be a frank review content of the consumer. The consumer inserts the review content and rating into the array of the “reviews” structures at the author’s address and the “datas” structure array of the metadata indexes in the structure. The transaction that calls this function also has a bytes32 type of metadata, a string type content and an integer type rating as input, and returns one if the transaction is successfully mined.

Further, check whether there is a review already made by the same user on the data by using the “existReview()” function. It returns one if a review exists; otherwise, it returns zero. The “getReview()” and “getRating()” functions also return the existing reviews or review ratings, and these three functions use the “constant” keyword. For the “setReview()” function, the result of the execution must be reflected in the blockchain database. Therefore, the user must wait until the transaction is created and consumes gas. However, the functions that validate values in a simple blockchain can be programmed to check only the return value using the “constant” keyword while designing a smart contract for efficiency.

A developer can extract the bytecode and ABI values by compiling with the Solidity conversion program in the completed smart contract. The bytecode value is the machine language value, and the ABI value is the interface value of the generated smart contract. We need to set the values in the JavaScript code as a variable and define the smart contract object using the Web3 API. To use a smart contract, it is necessary to deploy a transaction that creates a smart contract object so that the functions and structures of the contract can be used.

## 4. Analysis and Implementation

This section analyzes and compares the existing centralized review models with the proposed distributed smart contract-based review model. Additionally, we present the implementation results of our proposed model.

### 4.1. Comparison Analysis

**Figure 5:**
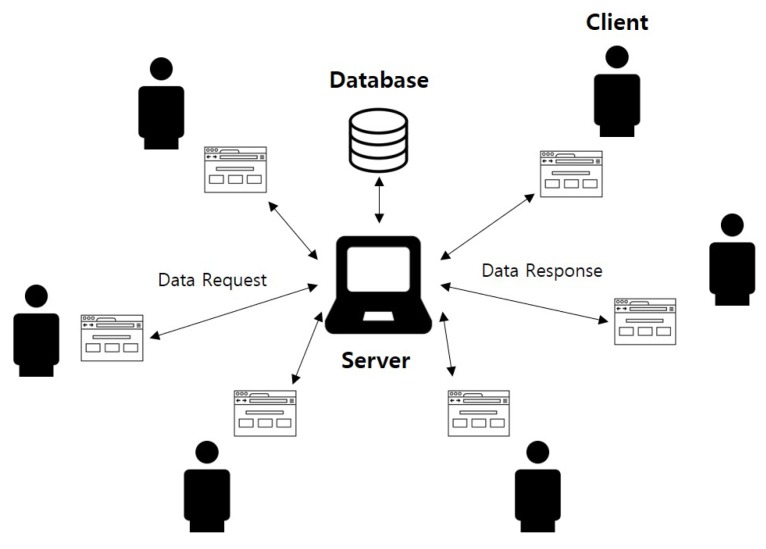
Structure of the centralized server-client model.

The existing review systems are mainly used by online retail and service providing platforms. The overall structure of the existing system is a centralized server-client model, in which a central server responds to numerous client programs requesting service, as shown in Figure 5. The server-client model has both an application and a network structure. This model separates the work between the client, which is the service requester, and the server, which provides the service. Generally, a server-client system refers to a system in which a computer runs an application that functions as a client making a service request, which is processed by the server. The server-client model is used in most web interfaces; thus, it is applied to systems with various purposes, such as customer support systems [21] and product data management systems [22], in addition to review systems. In [21], a customer support system using traditional methods, such as phone call surveys, is described as a method used by companies to receive customer feedback for improving the quality of their product or service. However, a web-based system has many advantages, such as an effective interaction between a customer and a system, and system accessibility; thus, the authors of [21] proposed a web-based customer support system. In [22], the advantages and disadvantages of the web-based server-client model were investigated in terms of their application to a product data management system. The advantage of a product data management system with a web interface is that this system is easy to use, access and connect to several subsystems. Conversely, the disadvantages of the client-server model consist of security vulnerabilities and bandwidth issues that occur when transferring large files, such as CAD files.

The operating process of a review system built using the server-client model is as follows. Users who wish to write a review will access the web page and register the review. The client program and the server program connected to the web page communicate with each other. The server handles the relatively simple process of storing the reviews in a connected database and sends the results to the client. The server-client web interface model has many related libraries and open source code; thus, less time and costs are required if a company wants to implement the model. A server processes the client requests immediately; thus, the request result can be applied quickly. However, the most serious drawback of this model is that all requests are handled only by a central server. This means that the server can be an SPOF, which is a security vulnerability for the system and may invite malicious actors to attack the system by using several methods, such as a man-in-the-middle attack, distributed denial of service attack, and so on. Additionally, if a network failure occurs between the server and the client, the client will not receive a request response, and the entire system will be incapacitated. Finally, in the existing review system, it is possible for users with elevated privileges, such as a server administrator, to delete reviews stored in the database. The purpose of the review system is to allow the user of the service to provide an honest evaluation of the service and thereby provide a reference to aid the decision-making process of other users. However, in practice, negative reviews of services or products can be removed by the service provider, which is unfair and lowers the overall reliability of the system.

**Figure 6:**
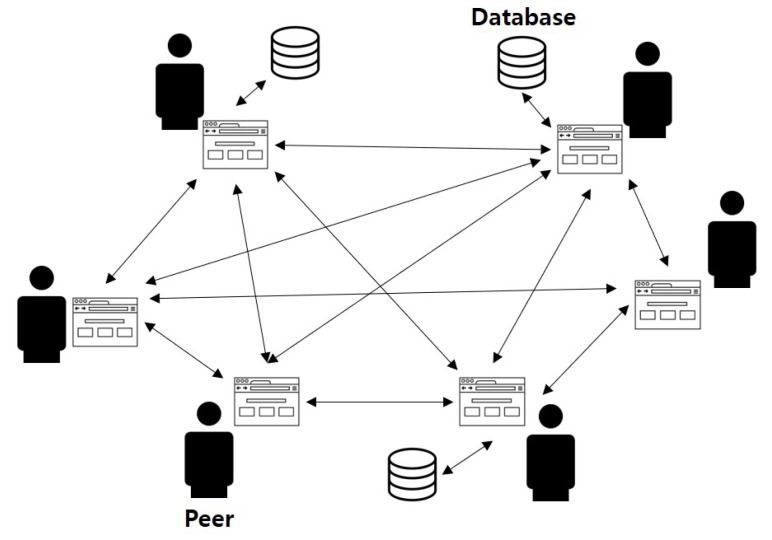
Structure of the decentralized blockchain-based model.

To overcome the disadvantages of the server-client model, the decentralized model has emerged, in which all nodes participate equally in the network, as shown in Figure 6. Moreover, the review system developed in this study uses a blockchain database based on the P2P network, which endows this model with the advantage of larger bandwidth, because all nodes in the network must communicate with each other. In most cases, the P2P network resources are typically distributed across multiple nodes in the network. The blockchain system also maintains a distributed database based on these characteristics. Even if some nodes are facing data sharing problems, other nodes can still receive data from the remaining nodes on the network. The blockchain-based model has the advantage of low maintenance cost. For a server to respond to all client requests, specific software and hardware are required to perform the request, which increases the cost of operation. However, in the blockchain-based model, which is a distributed P2P network environment, a single node is not expected to handle all tasks. Therefore, this model can be maintained at a relatively low cost.

Most of all, the review system based on the blockchain has advantages such as integrity, immutability and reliability, which are advantages of the blockchain. All reviews are stored in the decentralized blockchain database permanently; therefore, the review system provides immutability for once-registered reviews. All processes are transparently opened by cryptographic hash functions; thus, it is impossible for users to act maliciously. Our review system also provides reliability for malicious behavior, such as fake reviews; for example, if a user, who does not participate in the transaction, wants to write a fake review of the negative content for the purpose of economically damaging the data owner. In this system, however, the hash value of the IoT data transaction or metadata should be entered during the review registering process. Therefore, our system has a mechanism that anyone cannot write a review without participating in the transaction. Table 1 presents the advantages and disadvantages of both the server-client system and blockchain-based review systems.

### 4.2. Implementation Result and Performance

In this section, we describe the implementation result and performance of the review input length of the smart contract review model described in Section 3. The development and implementation were conducted on VMware Workstation 12. The implementation was programmed with the JavaScript, HTML/CSS and Solidity languages, and the implementation environment was Ubuntu 16.04 OS installed on a personal computer with 8 GB RAM and a quad-core CPU. The mining server and the Node.js interface run on the same virtual machine; thus, we did not consider network errors or bandwidth issues in the performance evaluation.

**Figure 7:**
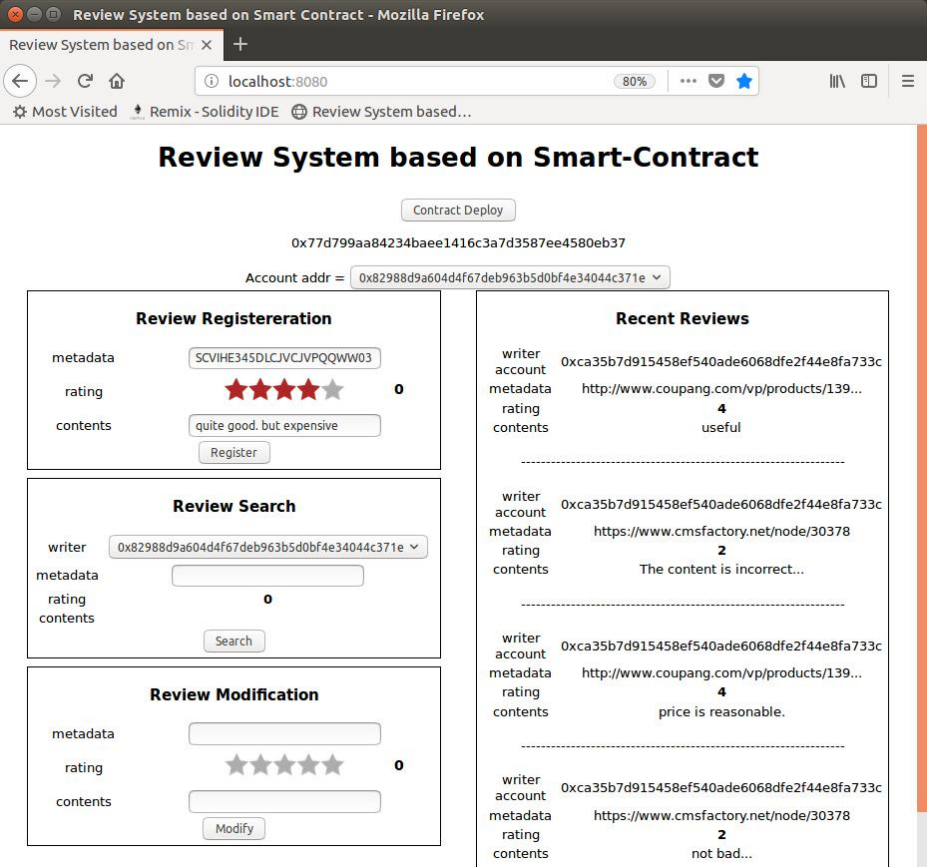
Developed HTML page.

Figure 7 shows the developed HTML user interface page. The web browser opens the HTML file linked to Node.js using port 8080. To create a new smart contract with the “Contract Deploy” button, the user must select the account that he/she wishes to use as his/her identity and enter the review items. The user must use the terminal to execute “geth.ipc” and unlock the account that he/she wishes to use. A new transaction is created by clicking the “Register” button. After a certain amount of time, the user can check the newly-added reviews in the “Recent Reviews” tab at the right side of page.

The smart contract used in this review system is a smart contract of the Ethereum platform. Ethereum smart contracts require a certain amount of gas to process a transaction. When a developer defines a smart contract entity, he/she also defines the total amount of gas to use. The amount of gas in the transaction is influenced by the number of codes to process and the input value. If the input value of the review registration transaction using the “setReview()” function is considered, three values are achieved for the metadata, rating and contents. The metadata are always 32 bytes in size because it uses the SHA256 hash function computed using the JavaScript code. The rating value is also four bytes in the int variable. However, the content variable is a variable length value because it is review content. That is, the amount of gas used in the transaction may vary depending on the length of the contents’ value. When we drove a smart contract-based review system, the longer the review length was, the more likely it would be mined, but not reflected in the chain. This was because the amount of gas used in the transaction exceeded 90,000, which is the base gas limit. To make up for this, we modified the JavaScript code in the UI page. In the JavaScript code, we created a smart contract object and arbitrarily increased the gas limit by adding the “gaslimit: 150,000” option value to “cont_obj.setReview.sendTransaction()”, which is a part of the registering review. To write a review that requires more gas, the developer must again modify the JavaScript code in the code UI page.

**Figure 8:**
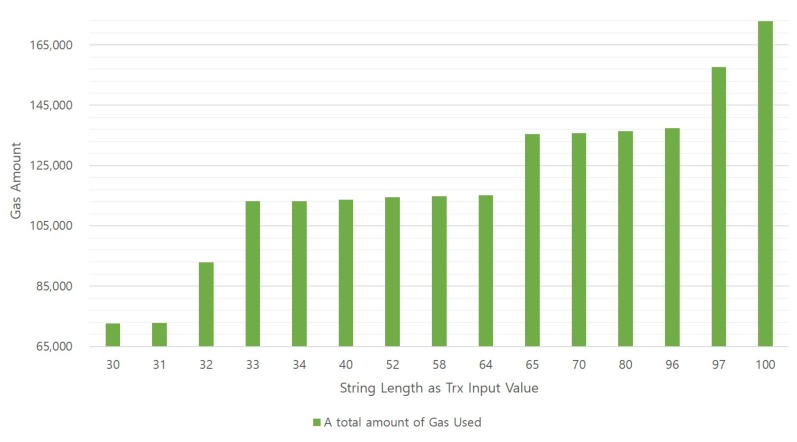
Change of amount of gas consumed with different input lengths of transactions.

**Figure 9:**
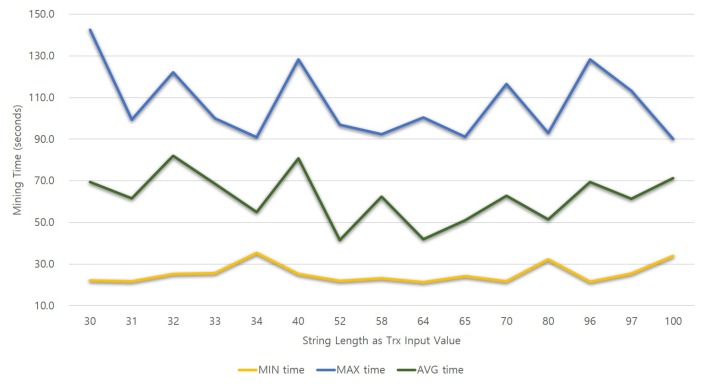
Change of mining time with different input lengths of transactions.

We conducted a test to determine the relationship between the length of the review content, amount of gas used and time required for successful mining. Our experiment uses an experimental design in which the independent variable is the string input length value of the transaction and the dependent variable is the gas amount and the mining time value required to process the transaction. The manipulation of the value of the independent variable is called a round, and in this experiment, it has 71 rounds in total. We also created 20 transactions for each round with the input of string values of the same length. As a result of the experiments conducting several rounds at the beginning of the experiment, the number of transactions to be generated in each round did not affect the experimental results significantly, measured and recorded spent gas values and times for each transaction. In this experiment, the values of 71 rounds were measured while increasing the string value from 30. Only 15 rounds showing meaningful values are shown in Figure 8 and Figure 9.

Figure 8 shows a graph of the relationship between the string input length value and the amount of gas used. The horizontal axis of the graph represents the input length value, which is an independent variable, and the green bar graph on the vertical axis represents the amount of gas consumed. The gas consumption per transaction is extracted using “web3.eth.getTransactionReceipt(TrxHash).gasUsed” of the Web3 API [23]. Figure 9 is a graph that illustrates the relationship between the string input length and transaction mining time. As shown in Figure 8, the result of the mining time value of the vertical axis according to the independent variable value of the horizontal axis can be confirmed in Figure 9. The time value is in JavaScript using “console.time()” and “console.timeEnd()” [24].

In the green bar graph in Figure 8, it can be seen that the larger the string length in the horizontal axis, the more gas is consumed. Transactions in the same round all consume the same gas, and the amount of gas consumed per round is different. Consequently, it can be seen that the length of the input value and the gas amount are proportional. In particular, when the number of string characters is 32 or more, the default gas limit of 90,000, which is an existing transaction, is exceeded, so the developer must correct the gas limit. We set the gas limit to 150,000, and we can write a review of up to 96 characters. An interesting result from this experiment is that there is no relationship between input length and mining time. The graph in Figure 9 shows the shortest time, the longest time and the average value of the mining time of 20 transactions in each round. In this graph, the average length of the mining time was the smallest in the test where the string length was 52; however, this seems to be temporary, which means even in the case of the longest string round, mining time is not related to the transaction size or the lines of code (LOC).

Based on the results of this experiment, we can assert that if a long string value is inserted into the transaction input value, a large amount of gas is consumed; thus, the developer must construct the system considering this and that users of this review system consume a gas fee proportional to their length. However, long length input values are not related to the mining time, because the mining time is influenced by the network or the miner’s choice. Thus, even if a user writes long reviews, the waiting time for mining can vary depending on the network conditions.

## 5. Conclusions

It is no exaggeration to say that human culture has developed with computers. Since the first attempt at combining clothing and computers in the 1960s at MIT, many scholars and companies have attempted to connect things around us with small computers and networks. Especially, the IoT technology is evolving rapidly and being applied in various fields, such as smart cities and smart homes, to provide comfortable living to people. Among these, a smart home refers to an IoT environment in which devices inside the house can communicate with the residents’ smartphones to transmit status information or control devices. Further, smart homes have the advantage that they can be easily accessed by people of different ages as compared to other fields. The investment potential and development potential are so great that many IoT device companies invest much money in technology and product development for a more innovative smart home. However, to develop products and technologies, the data accumulated by many users using the IoT devices are required. After analyzing the huge amount of log data stored by various users using the IoT devices, it is possible to know the shortcomings of these products or to develop new products with enhanced advantages. The desire for IoT development of companies has resulted in appearance of the useful IoT data sharing platform.

The IoT data marketplace is a platform for IoT data sharing where transactions occur between data consumers, who need data, and data owners, who want to sell their data. In recent years, many studies have considered a P2P marketplace model instead of the traditional server-oriented marketplace that has many drawbacks. Similar to the $S^{2}\mathrm{aaS}$ model in the IoT device environment, the decentralized data marketplace has a cloud storage, and the data owners only need to disclose metadata instead of the actual data. The quality of the actual data cannot be known from the metadata; thus, an IoT data review system is required to help the decision-making process of data consumers.

In this study, we developed and analyzed a review system for data traded in the IoT data marketplace. The metadata of the desired IoT data can be searched to determine whether to trade with a data owner based on the reviews submitted by the previous buyers. We performed various operations, such as creating a transaction using JavaScript, and developed a user interface with HTML. We refer to the ABI and bytecode values of the Solidity code in JavaScript, and use smart contract functions and structures. This is a blockchain-based system; thus, it is impossible for a server administrator to modify a review. Additionally, the proposed system ensures the integrity of the data and makes transactions, such as writing or submitting reviews, transparent to everyone to prevent malicious behavior. These characteristics of the block chain cause the proposed system to entail a certain amount of waiting period before the transaction is created. However, based on the results presented in Section 4, the input value of a transaction is not related to the time required for mining.

In future work, this system can be combined with a business model that encourages users to write reviews by incentivizing review submission. Ethereum smart contracts require an incentive for review submission because the transaction creator must pay for the gas to create the transaction. Therefore, an effective incentive mechanisms for the review submission process is necessary, and we expect that if the incentive mechanism is combined with our system, it can be applied to real IoT data marketplaces in the near future.

## Figures and Tables

**Table 1:** Comparison of the centralized model with decentralized model.

	Server-Client Review System	Blockchain-Based Review System
Network type	Centralized	Decentralized
Server required	required	not required
Network problem handling	Single point of failure	Connect another peer
	-> Entire system down	
Network features	Easier to implement	Less expensive system maintenance cost
	Typical model of web-based systems	Wider network bandwidth
Reliability and integrity	Existing reviews can be fabricated	Review database is maintained by blockchain
	-> The system is less reliable	-> No one can modify the reviews
